# Current Neurosurgery

**DOI:** 10.14789/jmj.JMJ23-0002-R

**Published:** 2023-03-29

**Authors:** AKIHIDE KONDO

**Affiliations:** 1 Department of Neurosurgery, Juntendo University Faculty of Medicine, Tokyo, Japan; 1 Department of Neurosurgery, Juntendo University Faculty of Medicine, Tokyo, Japan

**Keywords:** neurosurgery, brain tumor, endovascular surgery, functional surgery

## Abstract

Neurosurgery is based on neuroscience, physiology and medical physics. Therefore, neurosurgery has also developed along with discoveries and innovations in these fields. The present article outlines the areas of neurosurgery and their development until 2022.

Technology for the preservation of the central nervous system and cranial nerve function has made remarkable progress through the integration of diagnostic imaging and functional evaluation capabilities.

Endovascular treatment strategies of cerebrovascular disorders have also progressed. The procedures have not only shifted from craniotomy to endovascular catheterization, but the devices used in these procedures have also changed.

In addition to these traditional disease treatment strategies/techniques, neurosurgical techniques have recently been used in surgical procedures to improve quality of life. Epilepsy, is one of the diseases that does not significantly have a direct impact on life outcomes. However, epilepsy patients find it difficult to reintegrate into society. In epilepsy, seizure management is important, and some subgroups of patients can be better treated using surgical intervention than by using pharmacotherapy. In addition, the treatment of dementia due to idiopathic normal pressure hydrocephalus can be improved by surgical management of the cerebrospinal fluid. Neurosurgical intervention can help diseased patients reintegrate into society, which is difficult without treatment.

Even in these disease groups, surgical intervention may have irreversible consequences. Therefore, its implications should be decided based on universal scientific evidence.

## Introduction

Neurosurgery is a branch of neuroscience in which the surgeons aim to surgically treat diseases of the central nervous system (CNS). The CNS includes the brain and spinal cord, and the peripheral nerves that emerge from them. CNS surgeries must be performed only after they are validated on the basis of scientific, physiological, and physical theory.

Juntendo University has a Neurosurgery department that is operating for a long time and has dealt with complicated cases. One of the most significant examples is of Susumu Sato, the third president of Juntendo, who removed a depressed bone fragment and bullet to save a civil war survivor and also drained a brain abscess. This occurred approximately 100 years before neurosurgery was defined as a medical specialty in Japan.

Presently, the field has advanced and is progressing increasingly. The surgery performed by Dr. Sato previously can now be performed with less effort. The article provides a review of the recent trends in neurosurgery with focus on current challenges.

## The surgical removal of brain tumors

Brain tumor surgery is a technique that reflects recent technological innovations. Brain tumors are not necessarily the term for tumors of the brain parenchyma but often refer to intracranial neoplasms in general. Thus, it also includes lesions outside the brain parenchyma and neoplastic lesions in the pituitary gland and other organs.

For tumors located in the midbasal region of the skull (such as the pituitary gland), transnasal endoscopic surgery has been developed to reach the lesion through the nasal cavity that does not require craniotomy^1)^. Currently, the standard surgical approach for craniopharyngiomas and pituitary adenomas treatment is transnasal surgery. Additionally, this novel technique can be applied for the treatment of small meningiomas, chordomas of the clivus, and small lesions of the medial orbit^2)^. Previously, skull base surgery required knowledge of the anatomy of the skull base bone from above, as glimpsed through the space between the brain. Today, the skull base is viewed from below, and the knowledge required is changing.

**Figure 1:**
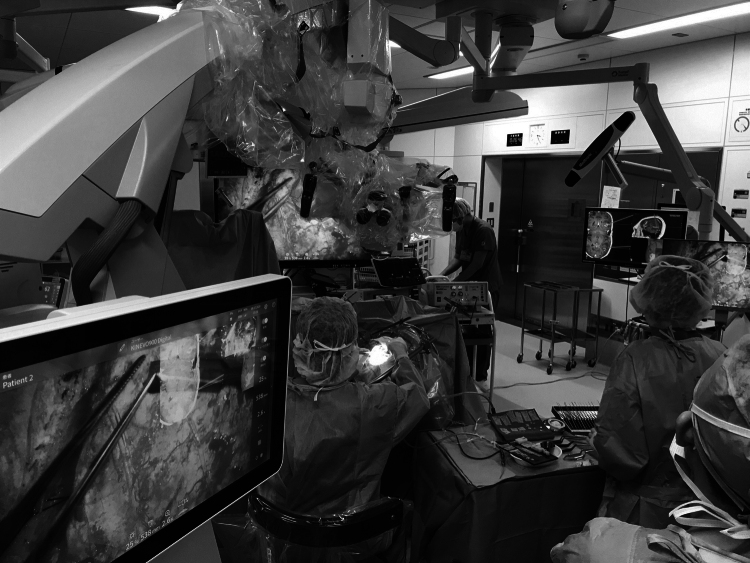
An operating room where brain tumor resection is performed. A number of monitors display various parameters to show the patient's functional preservation status.

Two major evolutions have taken place in the treatment strategies of parenchymal brain tumors: intraoperative neurological function preservation techniques. Since many parenchymal brain tumors are not benign, they cannot be curatively removed as they can infiltrate into functional brain. This is the reason why removal of the entire tumor from a functioning brain, regardless of its invasiveness, is detrimental to prognosis. Therefore, advanced methods are being developed to identify critical functional areas in preoperative images before surgery. Furthermore, it is now possible to perform intra-operative MRI with the cranium open and to use the information in real time while removing the tumor^3)^. This information is shared not only with the surgeon, but also to the medical staff. Therefore, current operating rooms resemble information processing rooms with countless monitors displaying various parameters as per the requirement (Figure 1).

The second is the diagnosis of brain parenchymal tumors. It is well-established that analysis of molecular biological characteristics of tumor cells help in accurate prognosis compared with conventionally used histopathology analysis (Table 1)^4)^. Thus, histopathology is no longer sufficient for the classification and diagnosis of brain parenchymal tumors. Many of these analyses are costly and require special techniques; therefore, it is essential to collaborate with research institute for analysis^5)^. In the future, there will be a need for the development of treatments based on these classifications.

**Table 1:** The list of diffuse gliomas in the 2021 World Health Organization Classification of Tumors of the Central Nervous System

Adult-type diffuse gliomas
Astrocytoma, *IDH*-mutant Oligodendroglioma, *IDH*-mutant and 1p/19q-codeleted Glioblastoma, *IDH*-wildtype
Pediatric-type diffuse low-grade gliomas
Diffuse astrocytoma, *MYB* or *MYBL1*-altered Angiocentric glioma Diffuse low-grade glioma, *FGFR*-altered Polymorphous low-grade neuroepithelial tumor of the young Diffuse low-grade glioma, *BRAF* V600E-mutant
Pediatric-type diffuse high-grade gliomas
Diffuse midline glioma, H3 K27M-mutant Diffuse hemispheric glioma, H3 G34-mutant Diffuse pediatric-type high-grade glioma,H3 wildtype and *IDH* wild type Bithalamic glioma, *EGFR*-mutant Infant-type hemispheric glioma, H3-wildtype

Modified from reference No.4.

## The procedure for vascular lesions

The changes observed in the treatment strategies of cerebrovascular disorders are as follows. First, transvenous thrombolysis was introduced for cerebral infarction or stroke cases. This therapy is designed to dissolve the thrombus as soon as possible after the onset of stroke, before endothelial damage progresses, thereby allowing the vessel to recanalize. Statistically, this has been shown to dramatically reduce stroke mortality. Furthermore, advances in cerebral infarction treatment have led to the development of devices that aspirate and retract thrombi from within blood vessels in cases of thromboembolization of major vessels. Mechanical thrombectomy, the physical removal of thrombi, is being introduced into the emergency settings. Since emboli in major cerebral vessels can disrupt a large area of the brain, removal of the thrombus can minimize the area of ischemia and improve functional prognosis.

In addition, many treatments for vascular lesions (cerebral aneurysms) have shifted from opening the skull and fissure of the brain to using intravascular catheters to reach the lesions^6)^. Technological innovations in cerebral aneurysms, in particular, have been remarkable. The conventional method of preventing aneurysm rupture, which is filling the aneurysm with multiple coils, has been replaced by the clinical use of a flow-diverter stent, which rectifies blood flow^7)^. This stent is inserted in the parent artery where the aneurysm has occurred, thereby preventing turbulent flow within the aneurysm. In addition, devices that can prevent re-rupture of a ruptured aneurysm such as a stent of a special shape that matches the diameter of the aneurysm, are also being used^8)^.

## Functional surgery

The main targets of neurosurgery are traumatic injuries, vascular disorders, and tumors, which, if untreated, can be fatal or disabling. This is because the nervous system, especially the CNS is vulnerable to external stress and it gets irreversibly damaged. Surgical treatment is considered to halt the progression of central nervous system disorders.

Recent advances in neuroradiological evaluation have made it possible to image intracranial and internal nerve conditions with considerable accuracy^9)^. This allows us to safely reach the site of injury and even depict temporary dysfunction due to compression or malalignment without causing major damage.

The field that has developed in this context is functional neurosurgery. It is a medical treatment that uses surgical procedures to improve the quality of life of patients affected with chronic illnesses that do not lead to death.

## Epilepsy

Epilepsy surgery is a representative of these treatment strategies. The prevalence of epilepsy is estimated to be 1 in 100 patients^10)^. More than 70% of these patients can manage their seizures and achieve social cure with medical treatment such as antiepileptic drugs. However, approximately 30% of the patients may require surgical treatment^11)^. Specific surgical procedures in this case include removal of the seizure focus to prevent of the progression of seizures. For example, when the seizure source is located in the medial temporal lobe, so-called generalized tonic seizures are secondary to complex partial seizures that are often awake but difficult to communicate with. This type of epilepsy is easily overlooked and requires special electroencephalography. In addition, because of the unique location of the medial temporal lobe, the rate of seizure control with antiepileptic drugs is significantly lower than that of other types of epilepsy involving the frontal lobe of the brain^12)^. The removal of the epileptic focus is the first-line treatment for this disease. Epileptic foci are scattered throughout the cerebrum on one side in epilepsy, causing secondary generalization that spreads seizures to the healthy brain via brain fibers. Weaning the fibers may reduce generalization and facilitate seizure management. In this case, dissection of the commissural fibers of the brain is indicated^13)^.

In addition, patients who are difficult to manage even with such surgery may be managed using vagus nerve stimulation, which is electrical stimulation of the cervical vagus nerve. In this procedure, electrodes are wrapped around the cervical vagus nerve and connected to a generator with adjustable electrical stimulation implanted in the chest to suppress severe seizures^14)^.

Patients who have had difficulty managing their epilepsy with medication and have had difficulty finding regular employment in their lives often return to normal life after undergoing epilepsy surgical procedures.

These surgeries have irreversible consequences on the central nervous system; therefore, careful determination of indications and preoperative analysis are important.

## Idiopathic normal pressure hydrocephalus

With recent medical advances, society is aging. Dementia is a disease that has attracted considerable attention. To date, no effective treatment for dementia has been found. However, some patients show mild cognitive symptoms, which occurs in a small percentage of patients due to hydrocephalus. This disorder is idiopathic with normal pressure hydrocephalus. Affected individuals present with mild attention and memory deficits, a unique wiggly gait, mild urinary urgency, and incontinence. Neurological symptoms can be ameliorated by draining cerebrospinal fluid through a tube into the abdominal cavity.

However, until now, this symptom has been the only diagnostic criterion, but our research group has identified a special protein that precipitates in the cerebrospinal fluid and found a method to diagnose the disease using this protein as a biomarker^15)^. In addition, we pointed out that the hydrocephalus-like appearance of enlarged ventricular diameters in this disease may be due to directional abnormality of ciliary motility in the ventricular ependyma^16)^. As a result, the diagnosis is now easier and patients can be expected to improve with the surgical tube placement. Patients who have achieved improvement in gait disturbance are pleased with the outcomes, proving that surgical treatment improves their quality of life.

## The other indications

Surgical treatment may have better outcomes than medical treatment. In cases such as hemifacial spasms and trigeminal neuralgia, where the symptoms are clearly caused by arterial compression of the causative nerve, surgical treatment is the only option. However, with advances in drug development, involuntary facial muscle movements and neuropathic pain caused by this compression can now be managed through drug administration, making drug therapy is the first choice. However, patients can be allergic to these drugs and prolonged use can render the treatment ineffective. Hence, there is a growing preference, both economically and psychologically, for surgical relief of the underlying cause of the compression phenomenon, rather than continuation of drug therapy^17)^.

Recent image analysis technology has made it possible to clearly delineate the positional relationship between nerves and blood vessels. In addition, the ease of identification of surgical pathways to lesions has dramatically improved the safety of surgery. This is one of the reasons why surgery and drug therapy can now comparable.

Other patient groups, such as those of Parkinson's disease and spinal cord disease, have better outcomes and prognoses with surgical intervention than with pharmacotherapy^18)^. Functional surgeries are now comparable to conventional treatment strategies.

## Conclusions

The present article compares the traditional (150 years ago) and contemporary neurosurgery methods (2000-2022). The field is advancing constantly. Although novel neurosurgery techniques are being developed, they are based on the basic principles of medical physiology and physics. The field will keep on developing along with evidence-based medicine. I believe that this article is the first one to provide a comprehensive information on both traditional and modern neurosurgeries.

## Funding

No funding was received for this manuscript.

## Author contributions

AK planned this manuscript, collected the appropriate literature information, and drafted the manuscript. AK also reviewed and approved the final version of the manuscript for publication.

## Conflicts of interest statement

The author declares that there are no conflicts of interest.
